# Fast Measurement of Methanol Concentration in Ionic Liquids by Potential Step Method

**DOI:** 10.1155/2015/106173

**Published:** 2015-01-31

**Authors:** Michael L. Hainstock, Yijun Tang

**Affiliations:** Department of Chemistry, University of Wisconsin Oshkosh, 800 Algoma Boulevard, Oshkosh, WI 54901, USA

## Abstract

The development of direct methanol fuel cells required the attention to the electrolyte. A good electrolyte should not only be ionic conductive but also be crossover resistant. Ionic liquids could be a promising electrolyte for fuel cells. Monitoring methanol was critical in several locations in a direct methanol fuel cell. Conductivity could be used to monitor the methanol content in ionic liquids. The conductivity of 1-butyl-3-methylimidazolium tetrafluoroborate had a linear relationship with the methanol concentration. However, the conductivity was significantly affected by the moisture or water content in the ionic liquid. On the contrary, potential step could be used in sensing methanol in ionic liquids. This method was not affected by the water content. The sampling current at a properly selected sampling time was proportional to the concentration of methanol in 1-butyl-3-methylimidazolium tetrafluoroborate. The linearity still stood even when there was 2.4 M water present in the ionic liquid.

## 1. Introduction

Methanol has the potential to be an efficient fuel for direct methanol fuel cells (DMFCs) with many applications ranging from small portable devices to large stationary power plants [1]. However, one major challenge that DMFC is facing is the fragility of the proton exchange membrane which is very thin in a DMFC. The thickness is typically less than 100 *μ*m [2]. A tiny crack or defect could fail the whole fuel cell. Ionic liquids (ILs) can be a replacement of the conventional proton exchange membrane to overcome the risk of fragility. Ionic liquids are composed of cations and anions. They are liquid at or near room temperature. Used as the electrolyte, ionic liquids possess many advantages such as high ionic conductivity, chemical stability, and resistance to high temperature. The current generation ionic liquids are stable even in the environment of moisture and air, making them ideal for general use as electrochemical media [3, 4]. In developing IL-based DMFC, the concentration of methanol must be controlled and monitored at several locations: the fuel feed, the electrolyte, the electrode assembly, and so forth. It is critical to develop a fast sensing technique for methanol in the environment of ionic liquids.

In this paper, we will report a simple and fast sensing technique for methanol. The technique is based on potential step analysis and is reliable even when water is present in the ionic liquids.

## 2. Materials and Methods

Chemicals and electrodes were purchased directly from the manufacturers or from venders such as Fisher Scientific and VWR: 1-butyl-3-methylimidazolium tetrafluoroborate (BASF quality, ≥98%) from Aldrich, methanol (reagent grade ACS) from Pharmco-AAPER, phosphate buffer saline (25X) from Thermo Scientific, L(+)-ascorbic acid (99%) from ACROS, potassium tetrachloroplatinate (II) 98% from Aldrich, sulfuric acid 2.0 N from LabChem, and gold disk electrode (2 mm dia.) from CH Instruments. The conductivity was measured with FiveEasy FE30 conductivity meter by Mettler Toledo. The potential step was performed with Electrochemical Workstation CHI650 by CH Instruments.

## 3. Results and Discussion

### 3.1. Sensing under No Water Conditions

Ionic liquids generally have high viscosity. 1-Butyl-3-methylimidazolium tetrafluoroborate (BMImBF_4_) is one of a few ionic liquids with relatively small viscosity, but it is still very viscous when compared to aqueous solutions. The presence of trace amount of less viscous methanol may reduce the viscosity or increase the conductivity significantly. Therefore, our most straightforward thinking was to sense methanol by the change in conductivity. Our thought was confirmed as the conductivity of BMImBF_4_ showed a linear relationship with the concentration of methanol in it.

**Figure 1:**
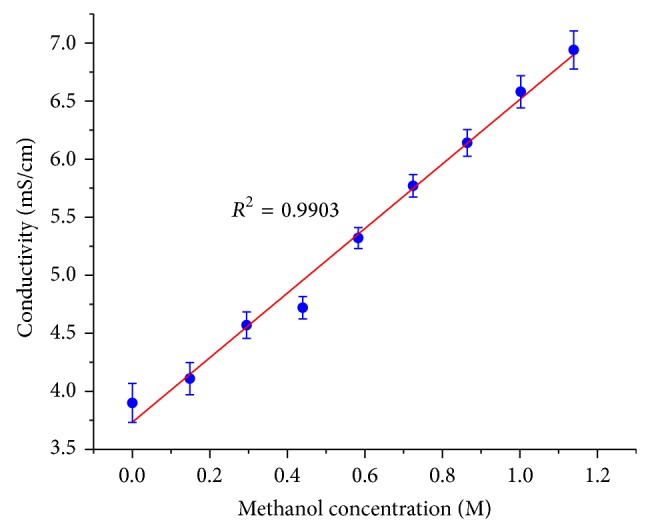
Conductivity of BMImBF4 changes with the methanol concentration in it. (The error bar indicates the 95% confidence intervals.)

As shown in Figure 1, the conductivity would be a good indicator of methanol content if there was no interference of water. However, the water is inevitable in a DMFC. Firstly, the oxidation of methanol requires the presence of water (1). Secondly, water is produced on the cathode and it could cross over to the electrolyte and to the anode (2). Thirdly, ionic liquids including BMImBF_4_ absorb water from the environment and the water content in BMImBF_4_ could reach as high as 0.29 wt% [5]:

(1) $$\begin{array}{c} \text{Anode:}\;\;\text{C}{\text{H}}_{3}\text{OH}+{\text{H}}_{2}\text{O}⟶\text{C}{\text{O}}_{2}+6{\text{H}}^{+}+6{\text{e}}^{-} \end{array}$$

(2) $$\begin{array}{c} \text{Cathode:}\;\;\frac{3}{2}{\text{O}}_{2}+6{\text{H}}^{+}+6{\text{e}}^{-}⟶3{\text{H}}_{2}\text{O} \end{array}$$

Water will also affect the conductivity of ionic liquids as reported before [6]. For this reason, the conductivity sensing method has little use in a DMFC. A practical method must be water-resistant.

### 3.2. Potential Step Sensing Method

We found a potential step method with Pt-nanoparticle-coated Au-nanoporous film (PGNF) was a reliable sensing method for methanol even when water was present. The construction of PGNF electrode has been reported earlier [7].

**Figure 2:**
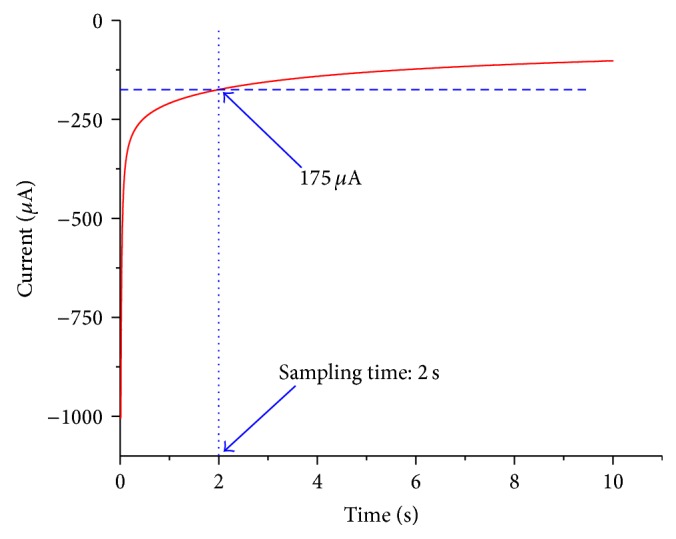
Chronoamperogram of a potential step measurement. 1.75 M methanol in BMImBF_4_. Step voltage: 1.9 V versus Ag/AgCl (saturated KCl).

Methanol was oxidized on PGNF electrode when the potential step was applied (1.9 V versus Ag/AgCl with saturated KCl solution). The decay of the current is shown in Figure 2. The current became smooth after 1 second and it was still measurable with amplitude of 175 *μ*A after 2 seconds. Our previous study has found that the adsorption/desorption and double layer charging were dominant within the first 2 seconds of potential step [8]. In the following report, the potential was fixed at 1.9 V versus Ag/AgCl (saturated KCl) and the sampling time at 2 seconds.

Under the small A/V (electrode area to electrolyte volume) condition and with a planar electrode, the current in a single potential step chronoamperometry can be predicted according to the Cottrell equation:

(3) $$\begin{array}{c} i\left(t\right)=\frac{nFAD_{0}^{1/2}C_{0}}{\pi^{1/2}t^{1/2}}. \end{array}$$

**Figure 3:**
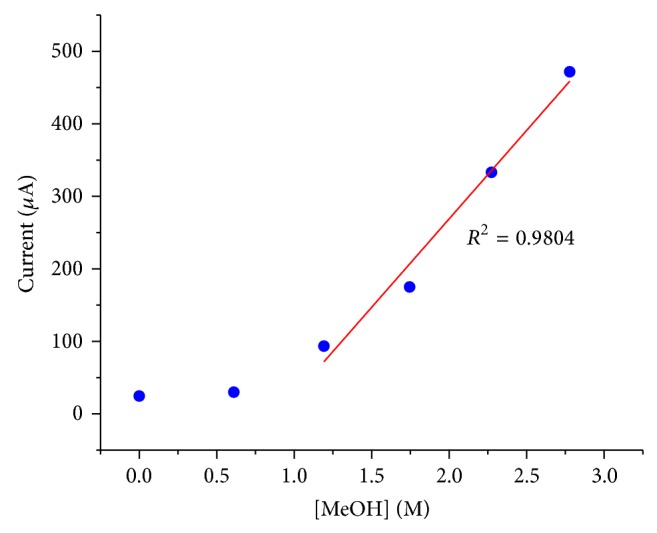
Current sampled at 2 seconds after the potential step versus methanol concentration in BMImBF_4_. Potential step: 1.9 V versus Ag/AgCl (saturated KCl).

In (3), *n* is the number of electrons involved in redox reaction, *F* is the Faraday constant, *A* is the electrode area, *D*_0_ is the diffusion constant, *C*_0_ is the bulk concentration, and *t* is the sampling time. If *t* is fixed in the potential step analysis, the sampling current *i*(*t*) should be directly proportional to the concentration *C*_0_ or the concentration of methanol in BMImBF_4_. The current at the sampling time (2 seconds) at various concentrations of methanol in BMImBF_4_ is shown in Figure 3.

The sampling current did not change too much when the methanol concentration was smaller than 1 M. Desorption/adsorption and double layer charging were predominant at the sampling time when the concentration was low [8]. When the concentration was above 1 M, the electrooxidation of methanol became the predominant process and the sampling current had a linear relationship with the methanol concentration.

**Figure 4:**
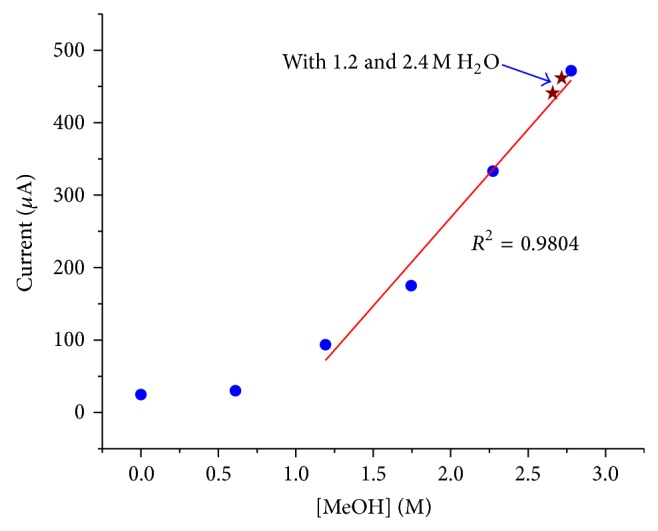
Two more points were added to Figure 3. Water was present in those two data points. The linearity still stood.

The potential step method described above not only had a good linearity but also was waterproof. When water was added to the methanol solution in BMImBF_4_, it did not interfere with the oxidation of methanol. The only effect of water was that it changed the molarity of methanol in BMImBF_4_. As shown in Figure 4, the linearity was not destroyed by the presence of water, even when the water content was as high as 2.4 M.

## 4. Conclusions

Conductivity monitoring was a reliable method in sensing methanol in ionic liquids, but its use was limited to the situation without the presence of water. A potential step method with properly selected potential step and sampling time could be a more practical method to analyze methanol concentration in ionic liquids especially when the methanol concentration was high so that the sampling current was predominantly contributed by the methanol oxidation.
